# The Effect of Equal-Channel Angular Pressing on the Microstructure, the Mechanical and Corrosion Properties and the Anti-Tumor Activity of Magnesium Alloyed with Silver

**DOI:** 10.3390/ma12233832

**Published:** 2019-11-21

**Authors:** Yuri Estrin, Natalia Martynenko, Natalia Anisimova, Diana Temralieva, Mikhail Kiselevskiy, Vladimir Serebryany, Georgy Raab, Boris Straumal, Björn Wiese, Regine Willumeit-Römer, Sergey Dobatkin

**Affiliations:** 1 Department of Materials Science and Engineering, Monash University, Melbourne 3800, Australia; yuri.estrin@monash.edu; 2 Department of Mechanical Engineering, The University of Western Australia, Nedlands 6907, Australia; 3 A. A. Baikov Institute of Metallurgy and Materials Science of the RAS, Moscow 119334, Russia; diana4-64@mail.ru (D.T.); vserebryany68@gmail.com (V.S.); dobatkin.sergey@gmail.com (S.D.); 4 National University of Science and Technology “MISIS”, Moscow 119049, Russia; n_anisimova@list.ru (N.A.); kisele@inbox.ru (M.K.); straumal@mf.mpg.de (B.S.); 5 N. N. Blokhin National Medical Research Center of Oncology of the Ministry of Health of the Russian Federation, Moscow 115478, Russia; 6 Institute of Physics of Advanced Materials, Ufa State Aviation Technical University, Ufa 450000, Russia; giraab@mail.ru; 7 Institute of Solid State Physics and Chernogolovka Scientific Center of the Russian Academy of Sciences, Chernogolovka 142432, Russia; 8 Institute of Materials Research, Division Metallic Biomaterials, Helmholtz-Zentrum Geesthacht (HZG), 21502 Geesthacht, Germany; bjoern.wiese@hzg.de (B.W.); regine.willumeit@hzg.de (R.W.-R.)

**Keywords:** biomedical materials, magnesium alloys, equal-channel angular pressing, microstructure, texture, mechanical properties, cytotoxicity in vitro

## Abstract

The effect of equal-channel angular pressing (ECAP) on the microstructure, texture, mechanical properties, corrosion resistance and cytotoxicity of two magnesium-silver alloys, Mg-2.0%Ag and Mg-4.0%Ag, was studied. Their average grain size was found to be reduced to 3.2 ± 1.4 μm and 2.8 ± 1.3 μm, respectively. Despite the substantial grain refinement, a drop in the strength characteristics of the alloys was observed, which can be attributed to the formation of inclined basal texture. On a positive side, an increase in tensile ductility to ~34% for Mg-2.0%Ag and ~27% for Mg-4.0%Ag was observed. This effect can be associated with the activity of basal and prismatic slip induced by ECAP. One of the ECAP regimes tested gave rise to a drop in the corrosion resistance of both alloys. An interesting observation was a cytotoxic effect both alloys had on tumor cells in vitro. This effect was accompanied with the release of lactate dehydrogenase, an increase in oxidative stress, coupled with the induction of NO-ions and an increase in the content of such markers of apoptosis as Annexin V and Caspase 3/7. Differences in the chemical composition and the processing history-dependent microstructure of the alloys did not have any significant effect on the magnitude of their antiproliferative effect.

## 1. Introduction

Development of magnesium alloys for application in bioresorbable implants is a burgeoning area of research [1,2,3,4,5,6]. Most of these works aim at the use of magnesium alloys in orthopedics and cardiovascular surgery. However, studies are becoming increasingly popular in which the ability of magnesium alloys to suppress the growth of tumor cells is scrutinized [7,8,9,10]. This opens up new possibilities for creating orthopedic implants or products with anti-tumor activity for insertion directly into the tumor area [11]. Obviously, the materials intended for such applications should, while possessing strong cytotoxicity towards tumor cells, not have a significant toxic effect on normal cells. Therefore, the alloying elements should be chosen carefully.

Among the currently studied magnesium alloys, Mg–Ag alloys appear to be promising [12,13]. Not only do such alloys have good biocompatibility but they also should have an antibacterial effect owing to the presence of silver [14]. However, alloying with silver is not sufficient for achieving the required level of mechanical properties. Therefore, additional strengthening is necessary if these alloys are to be used in orthopedic implants. For application of Mg–Ag alloys in wire products considered for direct insertion in the tumor area, their ductility needs to be sufficiently high. These targets can be achieved by severe plastic deformation (SPD) [15], equal channel angular pressing (ECAP) [16] being a particularly promising method. Thus, it is known that in addition to enhancing strength of metallic materials [17,18,19], ECAP may also lead to improvement of their in-service properties, such as corrosion resistance [20,21], fatigue life [18,22], wear resistance [23], electrical conductivity [24] and so forth. Improving the strength and in-service properties becomes possible due to the formation of the ultrafine-grained (UFG) structure and a profuse system of grain boundaries in the materials. In addition, there are cases of increased ductility of materials after SPD, as observed for example for magnesium [25,26,27] and aluminum [28,29] alloys. In the case of magnesium alloys, the formation of a specific texture in the ECAP process often leads to a situation where the ductility of the alloy increases but there is no significant strengthening, despite a pronounced grain refinement [30,31]. Therefore, studying the effect of the deformation parameters, particularly for ECAP as an example, on the structure, texture, mechanical properties and in-service performance of exemplary magnesium alloys is a matter of great importance. That is why the purpose of this work was to investigate the effect of the ECAP regimes on the structure, texture, mechanical properties and corrosion resistance of prospective medical magnesium alloys Mg-2.0%Ag and Mg-4.0%Ag, which are being targeted for use in the treatment of cancer. In addition, a study of the cytotoxicity and apoptosis (programmed cell death) these alloys to human tumor cells was conducted.

## 2. Materials and Methods

**Figure 1:**
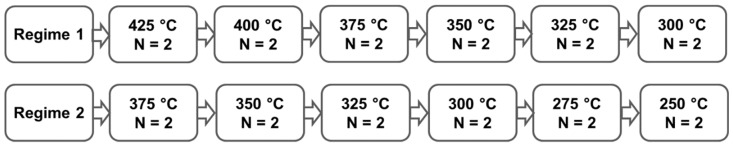
The equal-channel angular pressing (ECAP) processing regimes employed. (N denotes the number of passes at a given temperature.).

The alloys investigated, Mg-2.0(wt.)%Ag and Mg-4.0(wt.)%Ag, were obtained by smelting in a Nabertherm induction furnace (Nabertherm, Lilienthal, Germany) at a temperature of 720 °C using as a protective gas a mixture of Ar+ with 3 vol.% SF_6_. Then, cast ingots with a diameter of 60 mm underwent a T4 heat treatment (annealing at 450 °C for 8 h on Mg-2Ag and 16 h on Mg-4Ag followed by cooling by quenching in water). To obtain billets 10 mm in diameter required for further ECAP, the ingots of the alloys were extruded at a temperature of 425 °C with an extrusion ratio of 1:25 and a ram speed of 2.2 mm/s. The resulting condition will be referred to as the initial state of the alloys. Route Bc ECAP was carried out according to two different schedules, with temperature being dropped in discrete steps after defined strain increments (Figure 1). The intersection angle between the entry and exit channels of the ECAP die was 120° and the total number of passes was 12 for both ECAP regimes.

The microstructure of the alloys in the initial (extruded and heat treated) state and after ECAP was investigated using an optical microscope Axio Observer D1m (Carl Zeiss, Jena, Germany). Quantification of structural components was carried out by the method of random secants using the Image Expert Professional 3 software (Version 3, Moscow, Russia). Texture measurements were carried out using a DRON-7 X-ray diffractometer (SPE “Burevestnik,” St. Petersburg, Russia) in CuKα radiation with the aid of the Texx [32] and Texxor [33] software (Moscow, Russia). Six incomplete direct pole figures {10$\bar{1}$2}, {11$\bar{2}$0}, {10$\bar{1}$3}, {0004}, {20$\bar{2}$2} and {10$\bar{1}$4} were obtained with a maximum inclination angle α_max_ = 70° and a step size of 5° in the radial angle α and the azimuth angle β on a pole figure. The orientation distribution functions (ODFs) were calculated from the measured pole figures presented as a superposition of a large number (1000) of standard distributions with a small scatter. The centers of standard functions were located on a regular three-dimensional grid in the orientation space [33]. From these ODFs, complete pole figures were also calculated. The volume fractions of the major orientations were estimated using the ODFs as described in Reference [34]. Using the Euler angles and the volume fractions of the orientations, the generalized Schmid factors for the existing deformation systems and the inverse orientation factors were calculated, following the procedure reported in Reference [34]. The texture analysis was carried out in the direction parallel to the pressing axis. The mechanical properties of the alloys were evaluated based on uniaxial tensile tests carried out at room temperature in an Instron 3382 testing (Instron, High Wycombe, UK) machine with an extension rate of 1 mm/min. The tests were carried out on flat samples with a cross-section of 2 mm × 1 mm and a gauge length of 5.75 mm.

The mass loss measurements were carried out at a constant temperature of 37 °C and 5% CO_2_ atmosphere in a complete cell growth medium based on Dulbecco’s Modified Eagle’s Medium (DMEM) (Sigma-Aldrich, St. Louis, MO, USA), supplemented by 10% fetal bovine serum (FBS) (HyClon, Thermo Fisher, Loughborough, Leicestershire, UK), 2 mM glutamine, 100 mg/mL penicillin-streptomycin (all–PanEco, Moscow, Russia) after 2 and 7 days of incubation. At the end of each experiment, the samples were cleaned for 1 min in a 1000 mL cleaning solution (200 g Cr_2_O_3_ + 10 g AgNO_3_ + 20 g Ba(NO_3_)_2_ + reagent water balance) to remove the corrosion products. The change in mass was determined by weighing on an electronic scale GR 200 (A&D, Tokyo, Japan) with an accuracy of four marks per gram. The measure of the degradation rate, DR (expressed in mm/year), was calculated according to the following equation (ASTM_G1-03-E code) from the mass loss over 2 and 7 days:

$$DR=8.76\times 10^{4}\frac{\;\Delta m}{A\times t\times \rho }$$

where Δm is the mass loss in grams, t is the time of exposure in hours, A is the sample surface area in cm^2^ and ρ is the density of the alloy in g/cm^3^.

In order to study the cytotoxicity, the samples of both alloys in the initial state and after processing according to the second ECAP regime were sterilized by immersion in 70% ethanol for 18 h. Before the start of an experiment, the samples were pre-incubated in a complete growth medium overnight at 37 °C in an atmosphere of 5% CO_2_. Then the samples were placed one by one on the bottom of the wells of a 24-well plate (Corning Costar). As a cell model, human leukemia cells K562 line (collection of NN Blokhin NMRC of Oncology) were used. The choice of this non-adhesive cell line made it possible to conduct fast evaluation of cell reactivity without a need for trypsin treatment. It also allowed a quick assessment of the reactivity of cells without treatment by trypsin. The cells in logarithmic growth stage were re-suspended in a complete growth medium. 50 μL of a suspension containing 8.2 × 10^5^ cells was carefully added to each sample and to the bottom of the wells without samples (control). Plates with samples and cells were incubated for 15 min at 37 °C in an atmosphere of 5% CO_2_. Then, 1.95 mL of the complete growth medium was carefully added to the plate wells with cells, layering it along the walls of the well. Samples of the alloys were incubated with cells at 37 °C in an atmosphere of 5% CO_2_ for 24 h.

The cytotoxic effect of Mg-2.0%Ag (n = 3) and Mg-4.0%Ag (n = 3) alloys was assessed by studying the induction of lactate dehydrogenase (LDH) by cells in the culture medium after 2 h and 4 h of co-incubation. For this purpose, the cells in the wells with the samples and in the control were gently mixed by pipetting, then 100 μL of cell suspension were collected and centrifuged at 300 g. After that, 50 μL of the supernatant were collected. The measurements and analysis of the results were performed using the Pierce LDH Cytotoxicity Assay Kit (Thermo Fisher Scientific, USA) in accordance with the manufacturer’s instructions. Optical density was measured on an MS Multiscan plate reader (Labsystems, Thermo Fisher) using a 492 nm filter against 690 nm.

The studies of the number and characteristics of cells after incubation with Mg-2.0%Ag (n = 3) and Mg-4.0%Ag (n = 3) alloys were performed using the Muse Cell Analyzer and special kits (all–MD Millipore, Danvers, MA, USA) in duplicates in accordance with the manufacturer’s instructions. For this purpose, 250 μL of cell suspension after mixing was taken from each well after 2, 4 and 24 h of co-incubation with the samples. At least 2000 cells were analyzed for each test. Cell viability studies were performed using a Muse Count and Viability Assay Kit. Tumor cells apoptosis was investigated by determining the concentration of Annexin V(+) cells (using the Muse^®^ Annexin V and Dead Cell Assay Kit) and Caspase 3/7(+) cells (using the Caspase-3/7 Assay Kit). To assess oxidative stress, we measured the concentrations of ROS(+) cells and NO(+) cells inducing reactive oxygen species (ROS) in general and nitric oxide ions in particular (using Muse Oxidative Stress Kit and Muse Nitric Oxide Kit, respectively).

The Statistica 6.0 (StatSoft package; version 6.0, Tulsa, OK, USA) was used for statistical analyses. Comparative analysis was performed using Dunn’s test. Differences were considered significant at *p* < 0.05.

## 3. Results and Discussion

**Figure 2:**
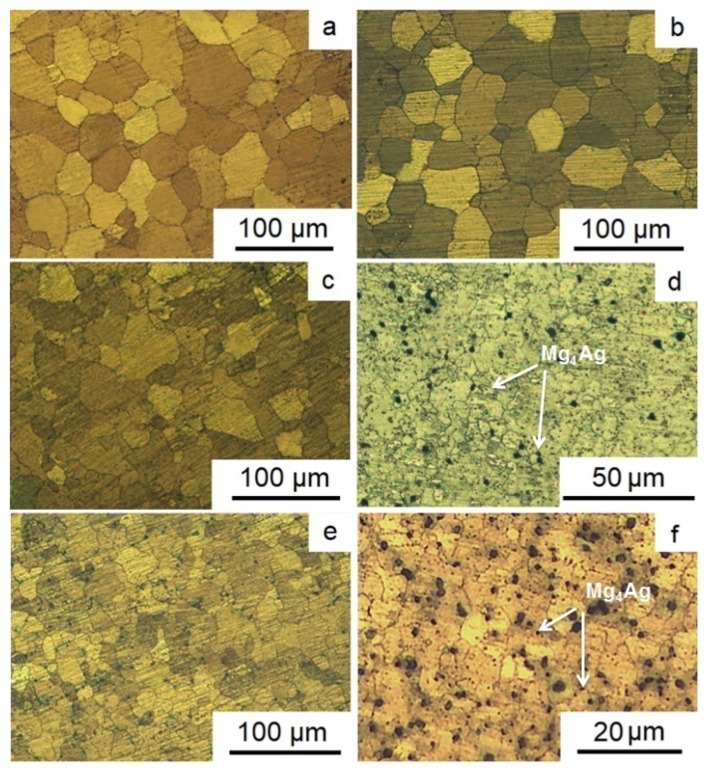
Structure of Mg-2.0%Ag (**a**,**c**,**d**) and Mg-4.0%Ag (**b**,**e**,**f**) alloys in the initial state (**a**,**b**) and after ECAP according to the first (**c**,**e**) and second (**d**,**f**) processing regimes.

Figure 2 presents the results of the study of the structure of Mg-2.0%Ag and Mg-4.0%Ag alloys in the initial state and after different regimes of ECAP. Extrusion at a sufficiently high temperature resulted in a structure of the initial state of both alloys consisting of almost completely recrystallized equiaxial grains of magnesium-based solid solution. For both alloys, no presence of a second phase after extrusion was detected. The average grain size for the initial state of Mg-2.0%Ag and Mg-4.0%Ag was 43.1 ± 5.1 µm and 41.2 ± 3.8 μm, respectively (Figure 2a,b).

ECAP processing of alloy Mg-2.0%Ag, for which the final deformation temperature was 300 °C, resulted in a refinement of the average grain size down to 23.9 ± 7.6 µm (Figure 2c). But the microstructure of the alloy in this case was quite heterogeneous, with both rather large grains some 50–60 μm in size and small grains of 8–10 μm in size being present. The reason for such a moderate grain refinement effect was the rather high deformation temperature, which promoted recovery processes and the concomitant annihilation of crystal lattice defects, including dislocations. As a result, high levels of dislocation density required for significant grain refinement were not accumulated even for true strains as large as 10, corresponding to 12 ECAP passes. In the case of the second ECAP regime, a lower final deformation temperature (250 °C) made it possible to refine the microstructure of the Mg-2.0%Ag alloy more strongly, down to the average grain size of 3.2 ± 1.4 μm (Figure 2d). In addition, a small number of second phase Mg_4_Ag particles were detected in this case [35]. Apparently, they precipitated by the decomposition of a supersaturated solid solution of silver in magnesium during heating and ECAP.

For the Mg-4.0%Ag alloy, the overall situation is similar. The first ECAP regime resulted in the refinement of the average grain size from about 41.2 ± 3.8 to 15.2 ± 4.8 µm (Figure 2e). The greater refinement of the Mg-4.0%Ag alloy structure, unlike the Mg-2.0%Ag alloy, is probably due to the large amount of silver in the composition. An increase in its content leads to a certain shift in the beginning and end of the recovery processes towards higher temperatures, which gives rise to a more significant grain refinement. The second regimes of ECAP leads to the refinement of the Mg-4.0%Ag alloy structure from 41.2 ± 3.8 μm to 2.8 ± 1.3 μm and precipitation of particles of the Mg_4_Ag phase (Figure 2f).

**Figure 3:**
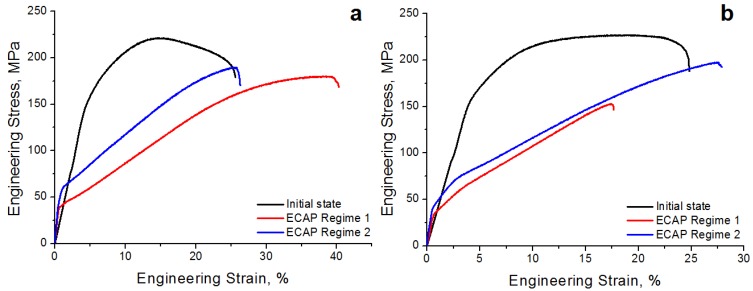
Engineering stress–strain response of Mg-2.0%Ag (**a**) and Mg-4.0%Ag (**b**) alloys before and after ECAP.

Table 1 and Figure 3 present the mechanical properties of the Mg-2.0%Ag and Mg-4.0%Ag alloys before and after both regimes of ECAP.

The ECAP of both alloys in both regimes, despite the grain refinement, leads to a slight decrease in the ultimate tensile strength. So in the case of the alloy Mg-2.0%Ag, the ECAP led to a decrease in the ultimate tensile strength from 220 ± 3 MPa in the initial state to 179 ± 1 MPa after regime 1 and to 182 ± 7 MPa after regime 2. For the Mg-4.0%Ag alloy, the situation is similar. The ultimate tensile strength of the initial state, equal to 225 ± 2 MPa, is reduced to 177 ± 33 MPa after regime 1 and to 204 ± 5 MPa after regime 2. However, decreasing the yield stress was more significant for both alloys after both regimes. So in the case of the alloy Mg-2.0%Ag, the ECAP led to a decrease in the YS from 147 ± 7 MPa in the initial state to 35 ± 5 MPa after regime 1 and to 53 ± 5 MPa after regime 2. The YS of the Mg-4.0%Ag alloy in the initial state is equal to 157 ± 6 MPa. ECAP leads to reduction of YS up to 35 ± 5 MPa after regime 1 and to 42 ± 5 MPa after regime 2.

At the same time, studies have also shown that the ECAP process leads to an increase in the ductility of the alloys. For the Mg-2.0%Ag alloy, the elongation increases from 17.2 ± 2.7% in the initial state to 34.0 ± 4.2 and 23.7 ± 0.6% after regimes 1 and 2 ECAP, respectively. For the Mg-4.0%Ag alloy, the elongation increases from 20.2 ± 0.3% in the initial state to 20.8 ± 7.4% after regime 1 and to 27.3 ± 2.1% after regime 2 of ECAP.

**Figure 4:**
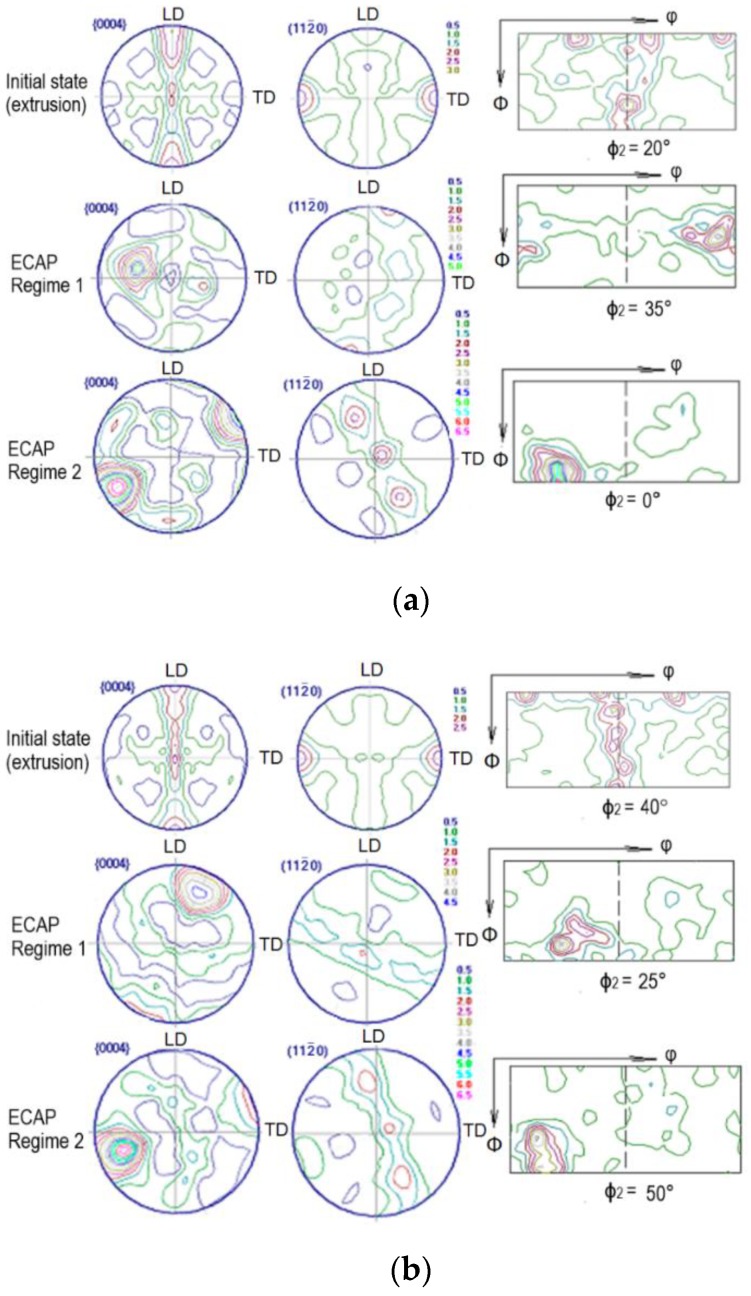
{0004} and {11$\bar{2}$0} pole figures and cross sections of orientation distribution function (ODF) of Mg-2.0%Ag (**a**) and Mg-4.0%Ag (**b**) alloys in the initial state and after ECAP (LD-longitudinal direction, TD-transverse direction).

To clarify the reasons for the slight increase in the strength characteristics combined with enhanced ductility, we investigated the texture of the alloys before and after ECAP. Figure 4 shows {0004} and {11$\bar{2}$0} pole figures and the ODF sections for Mg-2.0%Ag and Mg-4.0%Ag alloys in the initial state and after ECAP.

Table 2 shows the main orientations of the alloys in the initial state and after both regimes of ECAP. In the initial state of Mg-2.0%Ag alloy, weak primary orientations of the basal and prismatic type with a total volume fraction of 0.10 were observed. The volume fraction of non-texture component calculated for the longitudinal direction of texture analysis was high and amounted to 0.90. Under ECAP in the first regime, orientations of the inclined basal type were formed with a total volume fraction of 0.14. The fraction of the non-texture component slightly decreased to 0.86. The second regime of ECAP led to the formation of preferential orientations of inclined basal and prismatic types with a total volume fraction of 0.19. Accordingly, the volume fraction of the non-texture component dropped to 0.81.

The results for the Mg-4.0%Ag alloy were qualitatively similar to those for Mg-2.0%Ag. Thus, in the initial state weak predominant orientations of the basal and prismatic type with a total volume fraction of 0.12 were found (Table 2). The non-texture component calculated for the longitudinal direction was high in this case, too (0.88). After ECAP in the first regime, orientations of an inclined basal and prismatic type with a total volume fraction of 0.18 were formed and, accordingly, that of the non-texture component was 0.82. Again, the second regime of ECAP produced preferential orientations of the inclined basal and prismatic types, now with a total volume fraction of 0.17 (and a corresponding balance of 0.83 for the non-texture component).

The revealed preferential orientations after ECAP for both regimes had an effect on the tensile deformation behavior of both alloys studied through the activity of the operating deformation systems. This activity can be discussed in terms of the magnitude of their orientation factors, calculated by considering the volume fractions of the preferential orientations. Table 3 shows the values of the orientation factors for the main active slip and twinning systems for the alloys investigated.

From Table 3 it can be seen that, in the initial state, the activity of all deformation systems is approximately the same, with the exception of prismatic slip, for which the orientation factors are slightly higher than for other deformation systems. After regime 1 ECAP processing, the activity of the basal slip in Mg-2%Ag increases, while that of pyramidal slip and twinning decreases. This gives rise to a sharp texture corresponding to inclined basal slip (Figure 4a). As a result, the strength of the alloy drops relative to the initial state and its ductility nearly doubles, the tensile elongation rising from 17.2% to 34%. After regime 2 ECAP, the activity of prismatic slip increases, whereas that of basal slip declines. However, the texture corresponding to inclined basal slip is still detectable in the pole figures (Figure 4a). A probable cause for the different behavior of the alloy with the two processing histories may be a disparity of the magnitudes of the critical resolved shear stress for the basal and the prismatic slip. It is known that for magnesium the value of the critical resolved shear stress for the basal slip is lower than that for the prismatic slip. Therefore, a higher value of the orientation factor for the basal slip does not mean that it is completely suppressed. It is believed that a mixed texture can be formed in the alloy, comprising components corresponding to inclined basal slip and prismatic slip. In the Mg-4%Ag alloy, this trend appears after ECAP under both regimes (Figure 4b). The increased activity of the basal slip and the prismatic slip after ECAP of these alloys leads to enhanced ductility of these materials. In this case, one should remember that in all the pole figures {00.4} a tilted basal texture with a deflection angle of 50°–80° was found, which can cause a negative effect of ECAP on the strength characteristics. We observed this situation when studying the mechanical properties. Under both regimes tested, ECAP softened both alloys, despite a decrease in the average grain size. It should also be noted that the observed texture changes contribute to easy slip of dislocations during deformation.

**Figure 5:**
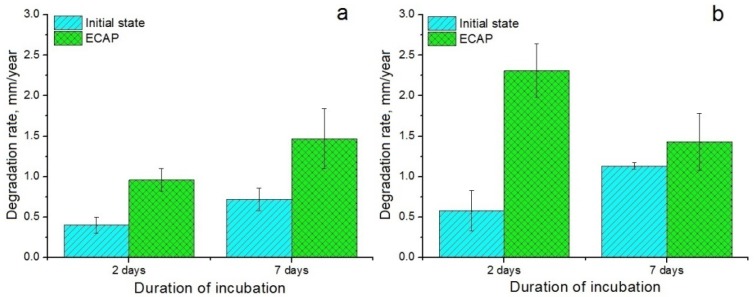
Degradation rate of Mg-2.0%Ag (**a**) and Mg-4.0%Ag (**b**) samples in the initial state and after ECAP processing (regime 2).

For medical magnesium alloys, in addition to strength characteristics, corrosion properties are also of great importance. Thus, for alloys intended for orthopedic implants it is necessary that the rate of degradation be sufficiently fast for the implant to gradually dissolve within a reasonable time. On the other hand, the biodegradation rate must be low enough for the implant to remain stable for the entire period of healing. Maintaining this delicate balance thus calls for tunability of the biodegradation process. For implant materials with antitumor activity, control of biodegradation rate governing release of metal ions in the tumor area and thus cytotoxicity to tumor cells, is a crucial requirement [11]. At the same time, it is important to limit the degradation rate to a level that would allow normal cells to adhere to the surface of the material and proliferate there. As shown in Reference [36], the optimal degradation rate of alloys of the Mg-Ag system is in the range of 1.5–2.2 mm/year. As mentioned above, in the present work, the bio-corrosion properties of alloys Mg-2.0%Ag and Mg-4.0%Ag were investigated by the mass loss method. Figure 5 presents the results of this study for these two alloys in the initial state and after ECAP processing (regime 2).

It can be seen from Figure 5 that ECAP leads to an increase in the biodegradation rate of both alloys as reflected in magnitude of the mass loss. In the case of Mg-2.0%Ag the increase in the degradation rate was not as substantial as in that of Mg-4.0%Ag. After two days of incubation, the degradation rate of the Mg-2.0%Ag alloy in the initial and ECAP-processed conditions was at a level of 0.40 ± 0.10 and 0.96 ± 0.14 mm/year, respectively. A longer incubation time (seven days), in general, did not change this tendency, although the degradation rate for both states of the alloy (0.72 ± 0.14 and 1.47 ± 0.37 mm/year, respectively) was more rapid. The situation was somewhat different in the case of the Mg-4.0%Ag alloy. After two days of incubation, the degradation rate of the alloy was increased by ECAP quite appreciably, from 0.58 ± 0.25 mm/year in the initial state to 2.31 ± 0.33 mm/year in the ECAP-modified condition. Incubation for seven days led to an increase in the degradation rate of the alloy in the initial state (to 1.13 ± 0.04 mm/year), while the degradation rate of the ECAP-processed alloy dropped substantially (to 1.43 ± 0.35 mm/year).

The observed decrease in the biocorrosion resistance of the alloys caused by ECAP is associated with an increase in the number of defects in the crystal structure and in the grain boundary area. No ECAP-induced formation of an ultrafine-grained structure was observed. An effect that had serious implications for the biocorrosion behavior of the alloys is the precipitation of the Mg_4_Ag phase particles and a depletion of the silver solid solution in magnesium that occurred during the ECAP process. In this case, the microstructure after ECAP comprised a silver-depleted matrix and particles of a more corrosion-resistant phase. This second phase, which is more resistant to corrosion, acts as a set of highly active microcathodes, which form numerous microcorrosive cells on the surface of the material. This gives rise to a faster degradation of the less corrosion-resistant matrix acting as an anode, which leads to an increase in the corrosion rate [13]. This statement is confirmed by the fact that the degradation rate of the ECAP-treated Mg-4.0%Ag alloy after the second day of incubation was significantly higher than that of the ECAP-treated Mg-2.0%Ag alloy, in which the amount of the second phase was noticeably lower. The observed decrease of the degradation rate of the ECAP-treated Mg-4.0%Ag alloy with the incubation time can be associated with the rapid formation of a protective layer of corrosion products. For the alloys in the initial state, this is not the case and a gradual degradation of the silver-oversaturated matrix occurs, with release of a larger amount of Ag+ ions to the surrounding culture medium than in the case of the ECAP-treated alloys. It should be noted that the above results are at variance with those for pure magnesium. Indeed, according to previous studies ECAP often does not reduce the corrosion resistance of Mg and can even slow down its degradation rate. Thus, it was shown [37] that, in contrast with the alloys investigated in this work, pure magnesium did not exhibit a deterioration of its corrosion resistance in a simulated body fluid (SBF). Also, it was shown in Reference [38] that a combination of hot rolling and ECAP can slow down the corrosion rate of pure magnesium relative to the cast state when tested in 3.5 wt.% NaCl solution. (The corrosion rate after 48 h of immersion tests was 0.054 and 0.025 mg·cm^−2^·h^−1^ for as-cast and deformed states, respectively). With regard to the Mg-Ag alloys studied here, it is encouraging that that with the right choice of the temperature conditions of ECAP, it is possible to improve the corrosion resistance in the alloys Mg-Ag.

As the alloys in question are being developed for use as materials with antitumor activity, in this case such an increase in the degradation rate with an increased ion release can be considered a positive factor. That is to say, the degradation rates (or the mass loss per time) obtained, despite an increase in magnitude, are acceptable for the application in mind. A natural aspect of this research was therefore a study of the interaction of the alloys considered with tumor cells. To that end, the level of LDH in the presence of specimens of the alloys, cell viability, Annexin V (+) cells and Caspase 3/7 (+) cells concentrations and the induction of NO- ions and ROS by tumor cells were investigated.

**Figure 6:**
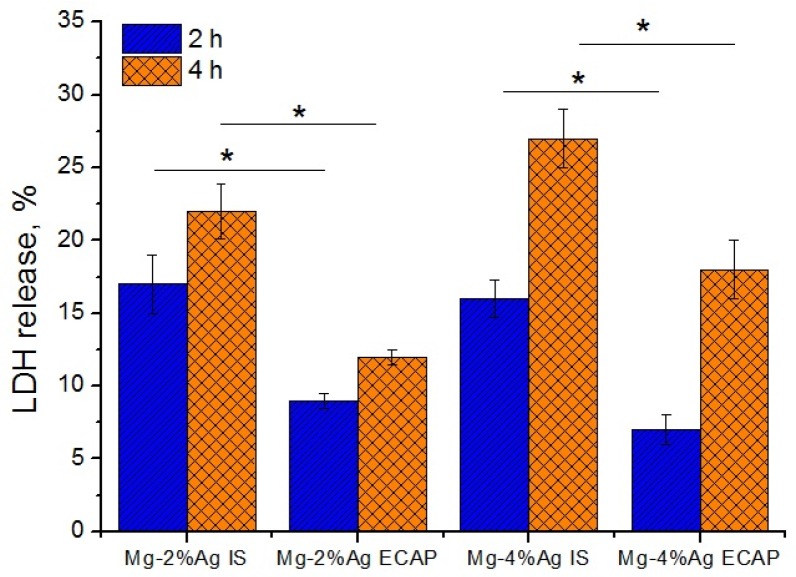
Increasing lactate dehydrogenase (LDH) level in the cell growth medium in comparison with the control (* *p* < 0.05). Time of incubation of tumor cells with alloys of Mg-2.0%Ag and Mg-4.0% Ag in the initial state (IS) and ECAP-treated (regime 2) state-2 and 4 h.

We evaluated the release of LDH from tumor cells after 2 and 4 h of incubation with samples to study the effect of the alloys on cell integrity. It was found that the LDH release of cells incubated with all types of alloys was increased in comparison with intact cells (control) already after two hours. Four hours after the start of the experiment, we observed a further increase in the LDH release, which indicates damage to the cell membranes induced by exposure to the tested samples. The LDH activity in the wells with samples of the alloys in the initial state was higher than in the wells containing samples of the ECAP-processed alloys. Samples of the alloy with a higher content of silver induced a greater LDH release in the culture medium (Figure 6).

**Figure 7:**
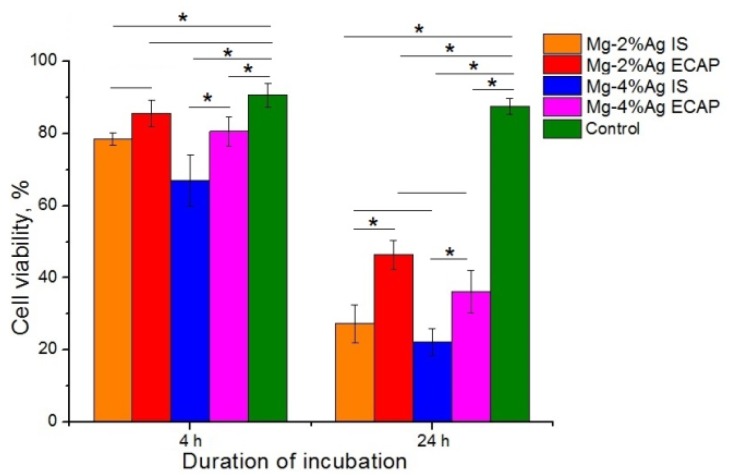
Effect of Mg-2.0%Ag and Mg-4.0%Ag alloys in the initial state (IS) and after ECAP (regime 2) on the viability of tumor cells (* *p* < 0.05).

An increase in the level of LDH is associated with damage to the cell membranes due to the cytotoxic effects of the alloys (Figure 7).

It was established with statistical confidence that the samples of the alloys in the initial state inhibited the viability of tumor cells more efficiently than ECAP-processed ones. Samples containing 4% Ag were found to be more toxic to tumor cells. The data obtained showed that both investigated alloys inhibited the viability of tumor cells already after 4 h of co-incubation. The smallest effect was exhibited by the Mg-2.0%Ag alloy processed by ECAP (regime 2). This type of samples inhibited the viability of tumor cells by only 5% (*p* > 0.05), while all other samples had a more pronounced, statistically significant effect: 12% for Mg-2.0%Ag in the initial state, 23% for Mg-4.0%Ag in the initial state and 10% for Mg-4.0%Ag after ECAP (calculated from the average values). This effect was even more pronounced after 24 h of co-incubation: 60% for Mg-2.0%Ag in the initial state, 41% for Mg-2.0%Ag after ECAP, 65% for Mg-4.0%Ag in the initial state and 52% for Mg-4.0%Ag after ECAP (calculated from the average values).

**Figure 8:**
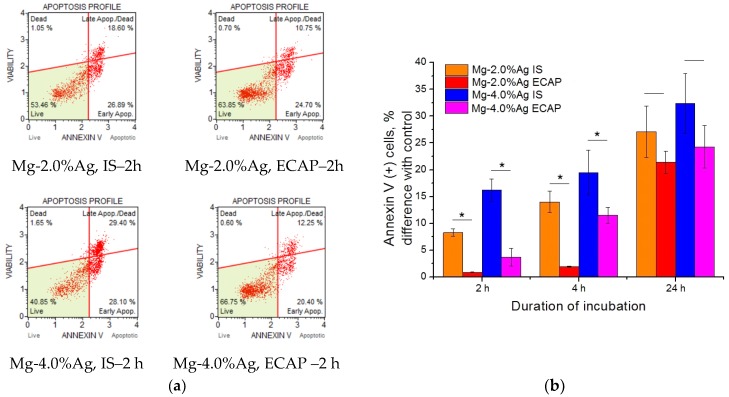
Increase of Annexin V (+) cells concentration after incubation with samples of Mg-2.0%Ag and Mg-4.0% Ag alloys in the initial state (IS) and after ECAP compared to the control (**a**) Annexin V(+) cells concentration; (**b**) difference with control) (* *p* < 0.05).

To unveil the mechanism of the detected cytotoxic effect, we studied the induction of apoptosis (programmed cell death) associated with increased expression of Annexin V cells and activation of Caspase 3/7 (Figure 8). It was found that incubation with alloy samples contributed to an increase in the concentration of Annexin V (+) cells, which may indicate an increase in the level of apoptosis in culture. Significant differences between the effectiveness of exposure to alloys in the initial state and the ECAP-processed one during the first days of incubation were observed—the activity of the former was markedly higher. This trend was also noticeable after 24 h but this was not statistically confirmed. Samples containing 4% Ag were more active than those with the corresponding processing history but a lower concentration of silver.

**Figure 9:**
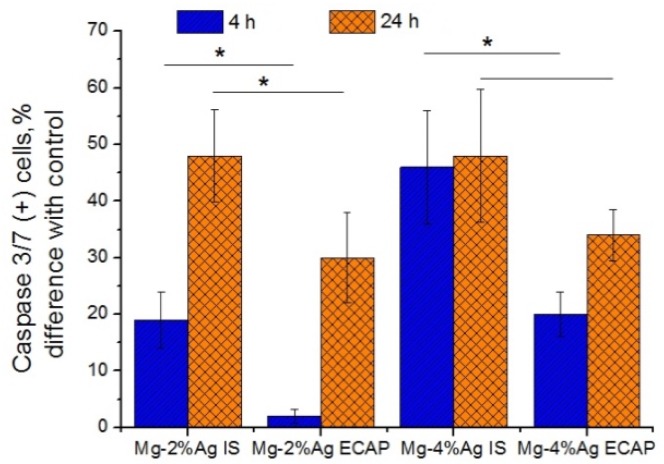
Increase of Caspase 3/7 (+) cells concentration after incubation with samples of Mg-2.0%Ag and Mg-4.0%Ag alloys in the initial state and after ECAP in comparison with the control (* *p* < 0.05).

Similarly, an increase of Caspase 3/7 (+) cells concentration after incubation with alloy samples was found (Figure 9). The samples in the initial state showed a greater effect than those in the ECAP-processed condition. After four hours of incubation, the effect of the leaner alloy, Mg-2.0%Ag, after ECAP did not differ significantly from the control, while contact with the other samples raised the number of Caspase 3/7 (+) cells in culture compared to the control—by 19% for Mg-2.0%Ag alloy in the initial state—by 46% for Mg-4.0%Ag alloy in the initial state and for Mg-4.0%Ag after ECAP in terms of the average values. The number of Caspase 3/7 (+) cells in the culture with alloy samples continued to grow with the incubation time—after 24 h the increment caused by the influence of the Mg-2.0%Ag alloy in the initial state amounted to 48% and of that after ECAP to 30% relative to the control. The corresponding values for alloy Mg-4.0%Ag were 48% and 34%.

Thus, it can be considered as established that the cytotoxic effect of both alloys on tumor cells is partially or fully governed by apoptosis, followed by activation of caspases in the tumor cells.

**Figure 10:**
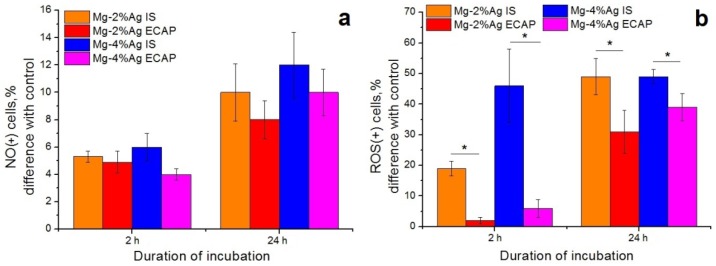
Production of NO ions (**a**) and reactive oxygen species (ROS) (**b**) by tumor cells during co-incubation with samples of Mg-2.0%Ag and Mg-4.0%Ag alloys in the initial state and after ECAP (regime 2, which is associated with oxidative stress in the cells in comparison with the control (* *p* < 0.05).

To clarify the mechanism of apoptosis initiated by tested alloys the effect of Mg-2.0%Ag and Mg-4.0%Ag on the tumor cells production of NO ions and ROS was studied (Figure 10).

The data presented in Figure 10 indicate an increase in the production of ROS and NO ions by tumor cells. This suggests that as a result of contact with alloy samples during co-incubation the cells develop an oxidative stress. However, the magnitude of this effect did not show any significant differences between samples in the initial and the ECAP-processed conditions.

It should be noted that the non-adhesive cell culture used made it possible to study the cells at different stages of their interaction with the Mg alloy specimens. Our data show that any of the tested alloy samples provoked a cytotoxic effect on tumor cells. Already two hours after the start of co-incubation, a violation of the structure of the cell membrane, which mediates an increase in the LDH release in the growth medium, was detectable. Further into the process, after four hours of co-incubation, a significant decrease in the tumor cells viability was observed. This trend was most pronounced under the influence of alloys Mg-2.0%Ag and Mg-4.0%Ag in the initial state, as documented quantitatively by a decrease in the viability of the culture by 12% and 23% and an increase in the level of extracellular LDH by 23% and 27%, respectively. These observations lead us to conclude that the investigated magnesium alloys, especially in the initial state, have a damaging effect on tumor cells causing their destruction during the first hours of incubation. The fact that the described cytotoxic effect was most prominent for samples of the alloys in the initial state, where more Ag+ ions were released into the medium during alloy dissolution, suggests that cell destruction was associated mainly with the increased concentration of silver ions in the culture.

It is likely that the release of metal ions in the process of biodegradation, along with a deterioration of the quality of the growth medium over time, triggers an apoptosis *pathway* in tumor cells, causing the antiproliferative effect of the alloys. This is supported by the observation that in the first four hours of co-incubation of tumor cells with Mg-2.0%Ag and Mg-4.0%Ag alloys in the initial state increased the expression of apoptosis markers Annexin V and Caspase 3/7 significantly more actively than was the case with the same alloys processed by ECAP. After 24 h of incubation, when the concentration of the released silver ions was equalized between the alloys in the initial condition and after ECAP due to accelerated degradation of ECAP-treated alloys, the difference in the efficacy of the alloys in these two conditions as anti-tumor agents decreased and in some cases was not statistically verified. It can be stated with reasonable confidence that apoptosis of tumor cells was due to the occurrence of an oxidative stress cascade and the production of ROS. It is known that an increase in the ROS level due to high reactivity leads to oxidative modification of biomolecules, changes in the activity of key enzyme systems, disruption of the membrane structure and damage to nucleic acids [39]. Our study showed a significant increase in the level of ROS in the culture of tumor cells caused by incubation with the tested Mg-Ag alloys. Furthermore, it was demonstrated that in the initial state these alloys had a greater effect on this process than their ECAP-treated counterparts. Thus, after four hours of incubation with the Mg-2.0%Ag alloy in the initial state, the concentration of ROS(+) tumor cells was 6.3 times higher than after the same time of incubation with the same alloy but processed by ECAP. After 24 h of incubation this ratio was 1.6. The effectiveness of the Mg-4.0%Ag alloy in the initial state exceeded that of the ECAP-processed alloy by a factor of 9 and 1.3 after 4 and 24 h of incubation, respectively. A decrease of the difference in the efficacy of samples with different structures with the incubation time can be explained by depletion of the cells redox potential and the reactivity of cells due to membrane destruction or by their programmed death, since most cells were already at some stage of apoptosis. Apoptosis most likely occurred due to the activation of redox-sensitive MAP-kinases. In the course of this study we found that, although the tested alloys increased the production of NO ions by cells, the release of these ions was not the main factor governing the intensity of the induced oxidative stress in tumor cells. This conclusion is also supported by the absence of noticeable differences in the concentration of NO(+) cells incubated with the tested alloys in both structural conditions (initial and ECAP-treated ones). There are therefore reasons to believe that the stimulation of the redox potential of cells by the tested alloys involves production of other aggressive oxygen ions. It is known that H_2_O_2_, O- and other ions [40] can play a key role in causing oxidative stress but identifying the specific governing agent for the alloy system considered would require further research.

According to the results reported above, alloys of the Mg-Ag system have the potential to be used in orthopedic products for the reconstruction of bone defects in cancer patients. For the treatment of this category of patients, it is necessary not only to ensure the functionality of the musculoskeletal system but also to reduce the risk of tumor recurrence or the occurrence of local metastases. While the results presented are encouraging, at present alloys Mg-2.0%Ag and Mg-4.0%Ag cannot be recommended for use in orthopedic articles, since their antitumor properties do not seem sufficient for achieving a significant therapeutic effect. The outcomes of this study suggest that magnesium alloyed with higher concentrations of silver or other elements (zinc-calcium, gadolinium, etc.) should be explored. In addition, extra deformation processing steps, for example, rotary swaging [41,42] carried out after ECAP may be a viable pathway to achieving the desired combination of properties. We intend to continue such investigations of the mechanical and biological properties of magnesium alloys, as well as the possibility of their modification in various ways. In particular, we are looking into ways to augment the alloys of Mg-Ag system with anti-tumor drugs. Also, it seems possible to produce wire with increased mechanical characteristics from the obtained materials due to the high ductility of the alloys processed by ECAP. Such a wire can be used for drug delivery to tumors. That is, the improvement of alloys using ECAP makes them potentially promising both for the development of orthopedic products for reconstruction of bone defects in cancer patients and for manufacturing systems for targeted drug delivery to tumors.

## 4. Conclusions

1. ECAP leads to a decrease in the average grain size, down to 3.2 ± 1.4 μm for the Mg-2.0%Ag alloy and to 2.8 ± 1.3 μm for the Mg-4.0%Ag alloy.
2. After ECAP of Mg-Ag alloys, a decrease in the ultimate tensile strength and the yield stress is observed. By contrast, the tensile elongation is raised, depending on the specific ECAP regime employed. Thus, for regime 1, the tensile elongation for Mg-2.0%Ag almost doubles—from 17.2 ± 2.7% in the initial state to 34.0 ± 4.2%. As a result of ECAP of Mg-4.0%Ag according to regime 2, the corresponding increase is from 20.2 ± 0.3% to 27.3 ± 2.1%.
3. The increased activity of the basal and prismatic slip promoted by ECAP leads to an increase in the ductility of the alloys studied, while the formation of a basal texture inclined by 50°–80° to the pressing direction causes a decrease in the strength characteristics.
4. ECAP according to regime 2 generally leads to an increase in the biodegradation rate. In the case of the Mg-2%Ag alloy, the degradation rate after 7 days of incubation was at a level of 0.72 ± 0.14 mm/year for the initial state and 1.47 ± 0.37 mm/year after ECAP. For the Mg-4%Ag alloy, the degradation rate grew in the early stages of incubation but then slowed down and remained unchanged within the measurement error. For this silver-rich alloy, the values of the degradation rate measured after 7 days of incubation were 1.13 ± 0.04 mm/year and 1.43 ± 0.35 mm/year for the initial state and after regime 2 ECAP, respectively.
5. Magnesium alloys with different structures, containing 2% and 4% silver, exhibit a cytotoxic effect on tumor cells in vitro, as was demonstrated for the K562 cell line. This effect is accompanied with the LDH release, an oxidative stress initiation and an increase in such markers of apoptosis as the Caspase 3/7 and Annexin V. Differences in structure and chemical composition had no significant influence on the antiproliferative effect of magnesium alloys. However, the alloys in the initial state inhibited the viability of tumor cells more effectively, which was correlated with the accelerated destruction of cell membranes leading to an increased release of LDH. In general, samples of the two Mg-Ag alloys investigated in this study had a greater anti-tumor effect, as signified by increased expression of Annexin V and the induction of ROS by tumor cells, when they were in the initial state, rather than in the ECAP-modified condition.

## Figures and Tables

**Table 1:** Mechanical properties of Mg-2.0% Ag and Mg-4.0% Ag alloys in various states.

Processing		YS, MPa	UTS, MPa	El, %
Mg-2.0%Ag	Initial state	147 ± 7	220 ± 3	17.2 ± 2.7
	ECAP, Regime 1	35 ± 5	179 ± 1	34.0 ± 4.2
	ECAP, Regime 2	53 ± 5	182 ± 7	23.7 ± 0.6
Mg-4.0%Ag	Initial state	157 ± 6	225 ± 2	20.2 ± 0.3
	ECAP, Regime 1	35 ± 5	177 ± 33	20.8 ± 7.4
	ECAP, Regime 2	42 ± 5	204 ± 5	27.3 ± 2.1

**Table 2:** The main orientations and their volume fractions for Mg-2.0%Ag and Mg-4.0%Ag alloys in the initial state and after ECAP. (The angles φ_1_, Φ and φ_2_ are the Euler angles and ω the volume fraction of the respective orientation.).

State of the Alloys		(hkil)<uvtw>	ϕ_1_	Φ	ϕ_2_	ω
**Mg-2.0%Ag**	**Initial state**	(0001)<6$\bar{1}\bar{5}$0>	7	0	0	0.02
		($\bar{1}5\bar{4}$4)<1$\bar{5}4.11$>	90	65	20	0.05
		(2.16.$\bar{18}$.5)<1$\bar{2}$.1.10>	84	82	35	0.03
	**ECAP Regime 1**	(19.$\bar{10}$.19)<$\bar{7}341$>	166	45	35	0.07
		(19.$\bar{10}$.19)<$\bar{16}$.2.14.7>	143	45	35	0.04
		(19.$\bar{10}$.13)<$7\bar{4}\bar{3}0$>	2	56	35	0.03
	**ECAP Regime 2**	($\bar{1}2\bar{1}$0)<50$\bar{5}4$>	36	90	0	0.10
		(03$\bar{3}$1)<5$\bar{5}\bar{2}\bar{3}$>	37	80	30	0.05
		($\bar{2}$.11.$\bar{9}$.11)<5$\bar{3}\bar{2}2$>	32	60	20	0.04
**Mg-4.0%Ag**	**Initial state**	(0001)<10$\bar{1}$0>	0	0	0	0.01
		(14$\bar{5}$.26)<$\bar{1}\bar{8}$93>	86	17	40	0.03
		(14$\bar{5}$3)<$\bar{1}\bar{1}$25>	95	71	40	0.02
		(15$\bar{6}$.12)<$\bar{1}\bar{6}$76>	88	39	40	0.02
		(14$\bar{5}$7)<$\bar{1}$.$\bar{10}$.11.14>	86	52	40	0.02
		(29.$\bar{11}$.0)<0001>	90	90	40	0.02
	**ECAP Regime 1**	($\bar{1}$.10.$\bar{9}5$)<3$\bar{2}\bar{1}3$>	46	75	25	0.06
		($\bar{1}5\bar{4}6$)<1$\bar{1}$01>	58	57	20	0.04
		($\bar{4}$.10.$\bar{6}3$)<2$\bar{1}\bar{1}4$>	67	78	5	0.04
		(45$\bar{9}$5)<$3\bar{5}$26>	60	70	55	0.04
	**ECAP Regime 2**	(23$\bar{5}$5)<9.$\bar{10}$.13>	20	60	55	0.06
		(24$\bar{6}$1)<7$\bar{6}\bar{1}$3>	25	84	50	0.04
		(02$\bar{2}$1)<6$\bar{3}\bar{3}$1>	10	75	30	0.04
		($\bar{1}4\bar{3}1$)<6$\bar{2}\bar{4}$3>	30	80	15	0.03

**Table 3:** Orientation factors for deformation systems of the alloys studied.

State of the Alloys		Basal {0001}<1120>	Prismatic {1010}<1120>	Pyramidal <c + a>	Twinning {1012}<1011>
**Mg-2.0%Ag**	Initial state	4.7	5.1	4.5	4.5
	ECAP Regime 1	4.1	5.2	5.0	5.4
	ECAP Regime 2	5.5	4.1	4.9	4.8
**Mg-4.0%Ag**	Initial state	4.8	5.3	4.4	4.4
	ECAP Regime 1	5.1	4.3	4.9	4.9
	ECAP Regime 2	4.9	4.4	4.9	4.9
